# Encephalomyocarditis virus infection in *Macaca sylvanus* and *Hystrix cristata* from an Italian rescue centre for wild and exotic animals

**DOI:** 10.1186/s12985-016-0653-9

**Published:** 2016-11-28

**Authors:** Giusy Cardeti, Valeria Mariano, Claudia Eleni, Marco Aloisi, Goffredo Grifoni, Stefania Sittinieri, Giampiero Dante, Valeria Antognetti, Efrem Alessandro Foglia, Antonella Cersini, Alberigo Nardi

**Affiliations:** 1 Istituto Zooprofilattico Sperimentale del Lazio e della Toscana “M.Aleandri” (IZSLT), Via Appia Nuova, 1411-00178 Rome, Italy; 2 Istituto Zooprofilattico Sperimentale del Lazio e della Toscana “M.Aleandri” (IZSLT), Viale Europa, 30-58100 Grosseto, Italy; 3 Centro Recupero Animali Selvatici della Maremma (CRASM) e Animali Esotici (CRAE), SO 34 km 3,5, 58055 Semproniano, Grosseto, Italy; 4 Istituto Zooprofilattico Sperimentale della Lombardia e dell’Emilia Romagna “Bruno Ubertini” (IZSLER), Via A. Bianchi, 7/9-25124 Brescia, Italy

**Keywords:** Encephalomyocarditis virus, Barbary macaque, Crested porcupine, Outbreak, Italy, Animals, Wild, Captive

## Abstract

**Background:**

The Encephalomyocarditis virus (EMCV) is a small, non enveloped, positive sense single-stranded RNA virus in the genus *Cardiovirus,* family *Picornaviridae*, with two known serotypes. It is spread worldwide and infects a huge range of vertebrate hosts with zoonotic potential for humans. The pig is the mammal most likely to be impacted on with the disease, but EMCV occurrence has also been reported in non-human primates and in a variety of domestic, captive and wild animals. Until now, human cases have been very rare and the risk appears to be almost negligible in spite of human susceptibility to the infection.

**Case presentation:**

Between September and November 2012 a fatal Encephalomyocarditis virus outbreak involving four Barbary macaques and 24 crested porcupines occurred at a rescue centre for wild and exotic animals in Central Italy. In this open-field zoo park located near Grosseto, Tuscany about 1000 animals belonging to different species, including various non-human primates were hosted at that time. Sudden deaths were generally observed without any evident symptoms or only with mild nonspecific clinical signs. The major gross change was characterised by grey-white necrotic foci in the myocardium and the same EMCV strain was isolated both in macaques and crested porcupines. Phylogenetic analysis has confirmed that only one EMCV strain is circulating in Italy, capable of infecting different animal species.

**Conclusions:**

This report confirms the susceptibility of non-human primates to the EMCV infection and describes the disease in porcupine, a common wild Italian and African species. No human cases were observed, but given the zoonotic potential of EMCV these findings are of importance in the context of animal-human interface.

## Background

The Encephalomyocarditis virus (EMCV) is a small, non enveloped, positive sense single-stranded RNA virus in the genus *Cardiovirus,* family *Picornaviridae*, with two known serotypes [1]. A probable third EMCV type has been isolated from orang utan in 2001 and 2002 [2]. It is spread worldwide and infects a huge range of vertebrate hosts with zoonotic potential for humans [1, 3, 4].

The pig is the mammal most likely to be impacted on with the disease [5, 6], but EMCV occurrence has also been reported in non-human primates and in a variety of domestic, captive and wild animals. Several lethal outbreaks have been described in zoos in Australia and the USA [2, 7, 8] and in Northern Italy [3].

EMCV can cross the species barrier [3] and the possible pig-to-human transmission by xenotransplantation has increased the interest in this virus. Fortunately until now, human cases have been very rare [9] and the risk appears to be almost negligible in spite of human susceptibility to the infection.

## Case presentation

This study describes the occurrence of EMCV infection in macaques and crested porcupines housed in a WWF Rescue Centre for Wild and Exotic Animals, in Central Italy. The Centre is 20 ha in size and at the time of the epidemic hosted about 1000 animals belonging to different species including various primates, felids, reptiles, parrots and birds of prey, mainly seized by Police Authorities because of illegal detention.

The disease outbreak occurred between September and November 2012, when four Barbary macaques (*Macaca sylvanus*) and 24 crested porcupines (*Hystrix cristata*) died.

The macaques (*n* = 22) were housed in three different external hutches with seven or eight animals in each; the crested porcupines (*n* = 25) were housed in a hutch within a greenhouse and about 800 m from the macaques.

Between the end of September and the end of October four primates died after 12 to 24 h of mild, nonspecific clinical signs including lethargy and weakness. Shortly afterwards, the crested porcupines became infected and by mid November, 24 of the 25 subjects were found dead over a period of about a week.

Neither symptoms nor abnormal mortality were observed in other animal species at the Centre.

Necropsies were carried out on all macaques and on 17 of the 24 crested porcupines. For the histopathological examinations, the samples were fixed in 10% buffered formalin, embedded in paraffin, cut at 5 μm and stained with haematoxylin-eosin. Parasitological and bacteriological examinations were performed on selected internal organs according to standardized protocols. Liver and gastric content samples from three porcupines were submitted for toxicological exams to detect pesticides and methaldehyde.

For virological examination lung, spleen, liver, brain and heart were processed and separately inoculated into VERO (African green monkey kidney) cells and BHK21 (Baby hamster kidney 21) cells [10]. Cell culture lysates and the intestinal content of all porcupines were investigated by negative staining immunoelectron microscopy (IEM). A positive reference serum produced by Istituto Zooprofilattico Sperimentale della Lombardia e dell’ Emilia Romagna (IZSLER) against Novara 86 strain of EMCV was employed using the Airfuge method [3].

Finally, biomolecular analysis was performed on samples from both *Macaca* and *Hystrix*. After RNA extraction and reverse transcription, a PCR End Point protocol [11] was used targeting a 285bp region internal to 3’ conserved end of viral polymerase (3D). The 285bp PCR products were purified and sequenced in an automated sequencer. The obtained nucleotide sequences were analysed and compared to those published in the GenBank database (https://www.ncbi.nlm.nih.gov/genbank/) using the Nucleotide Blast (BLASTn) program. For virus typing the complete P1 (capsid-encoding) gene region was amplified and sequenced. Viral RNA extraction was performed on samples both from macaque and porcupine using the commercial kit QIAamp viral RNA (Qiagen). The VP1 coding sequences were amplified by One Step RT-PCR kit (Qiagen) and using two specific primers (EMCVff2 5’-ACCTCAGCCAAGATCCTTACA-3’; EMCVrev1 5’-CTCAGAATCACGTCCGCA-3’). A portion of 1070 bp was sequenced using Big Dye terminator Kit (Applied Biosystems) with the same primers used for amplification, and with two internal primers in addition (EMCVffint3 5’-GGCTTTGCACCTTTCTCCAA-3’; EMCVrevint3 5’-GCTTCGTCCCTGCAAAGAAA-3’). The obtained sequences were assembled and edited (Lasergene, DNAstar). Phylogenetic tree was generated with Neighbour Joining algorithm with Kimura-2 parameters and Bootstrap 1000 repeats phylogenetic test. Alignment and phylogenetic analysis were made by MEGA6 software.

**Fig. 1:**
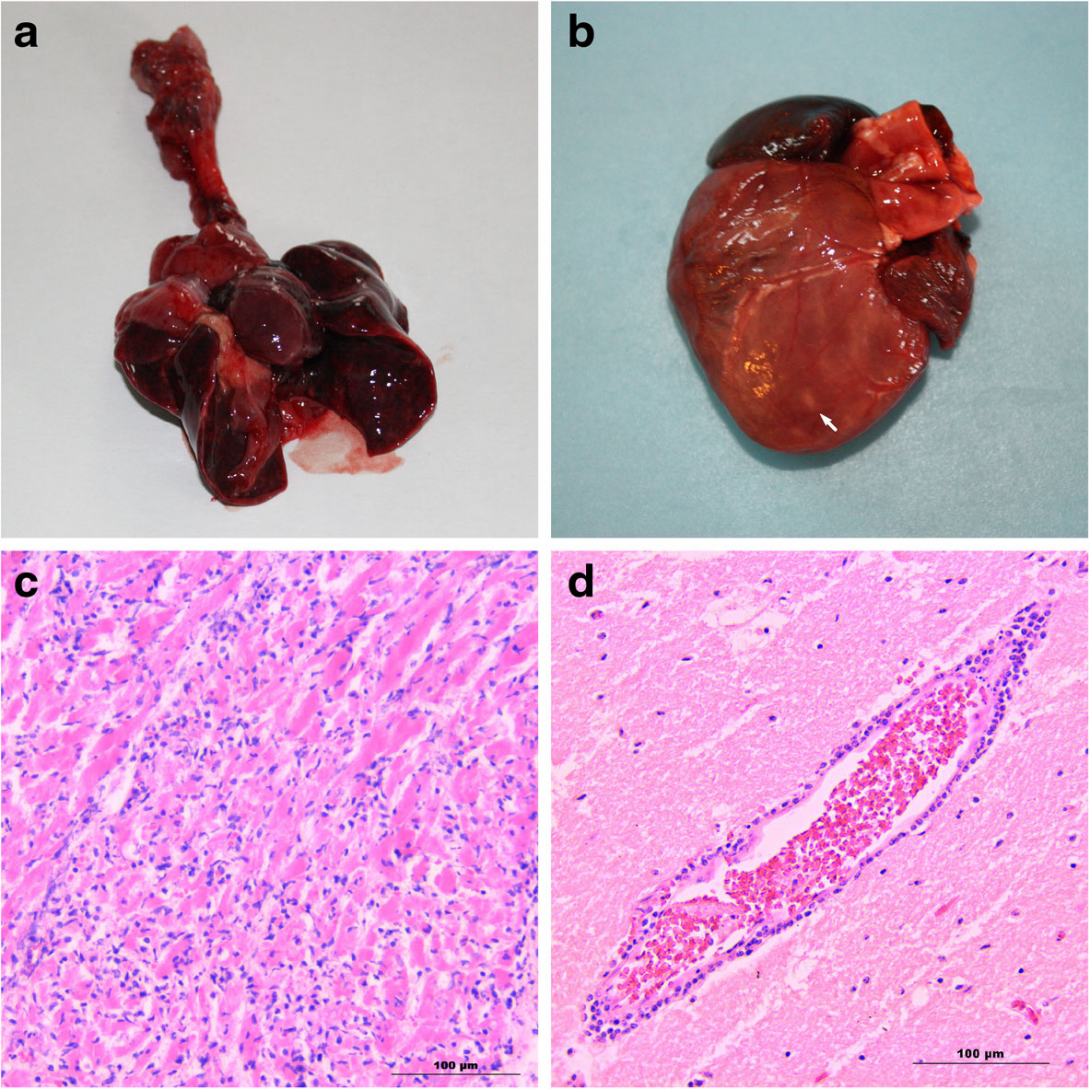
Main gross and histological findings in macaques and porcupines. **a** Lungs of a *Macaca sylvanus*. Severe congestion and oedema. **b** Heart of a *Hystrix cristata*. Whitish foci throughout myocardium (*arrow*). **c** Heart of a *Macaca sylvanus*. Lymphocytic and neutrophilic interstitial myocarditis. Bar = 100 μm (haematoxylin-eosin). **d** Brain of a *Hystrix cristata*. Lymphocytic perivascular cuffing. Bar = 100 μm (haematoxylin-eosin)

During necropsy, similar lesions were observed in both macaques and porcupines (Table 1; Fig. 1a, b). Some nematodes were found in the intestine of one porcupine. Typical histological lesions were confined to the cardiovascular system and in porcupines also to the brain (Table 1; Fig. 1c, d).

**Table 1:** Main gross and histological findings observed in EMCV outbreak

Species	N° of animals	Gross pathology^(a)^	Histological findings^(a)^
*Macaca sylvanus*	4	Sero-haemorrhagic pleural and pericardial fluid (4), pulmonary oedema and congestion (4), liver congestion (4), kidney congestion (3), meningeal congestion (3)	Severe lymphocytic and neutrophilic interstitial myocarditis, with areas of necrosis (4/4), congested organs (4/4)
*Hystrix cristata*	17	Sero-haemorrhagic pleural and pericardial fluid (11), sero-haemorrhagic peritoneal fluid (6), pulmonary oedema and congestion (10), cardiomegaly and grey-white necrotic foci in the myocardium (5), meningeal congestion (2)	^(b)^Severe lymphocytic and neutrophilic interstitial myocarditis, with areas of necrosis (9), multifocal lymphocytic meningoencephalitis (6), pulmonary congestion (9)

^(a)^In parentheses the number of animals showing the described lesion. ^(b)^Histological examination was performed on n.9 *Hystrix cristata*

Bacteriological investigations evidenced bacteria of secondary infection and toxicological analysis were negative.

**Fig. 2:**
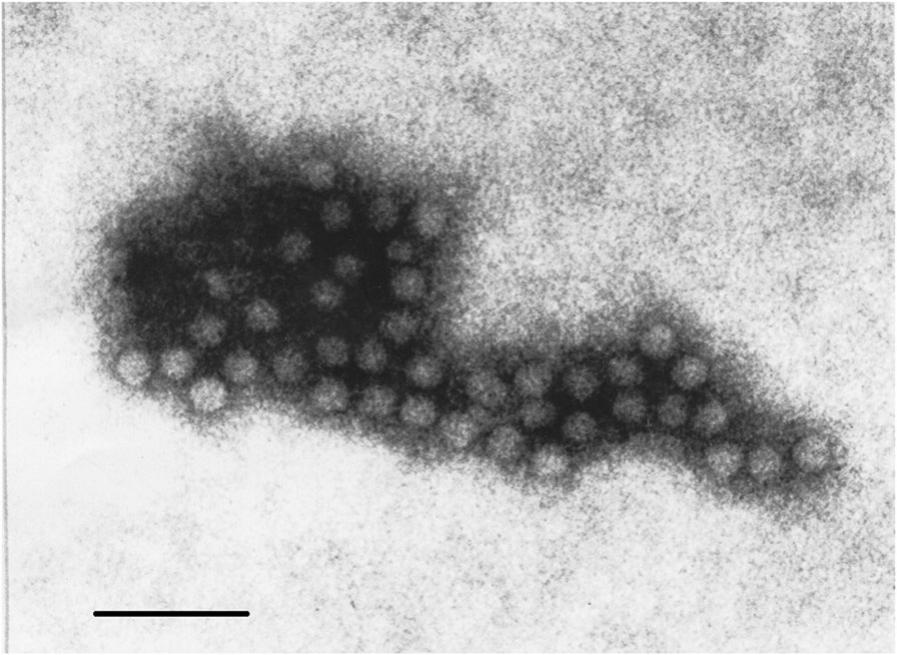
Electron micrograph. EMCV particles using IEM. (2% PTA. Bar = 100 nm)

Virus was isolated in all sampled organs from macaques and crested porcupines. VERO and BHK21 cell cultures showed a cytopathic effect after 48–72 h post-inoculation of homogenates of different organs. The virus was identified as EMCV by using the IEM technique (Fig. 2).

Aggregates of EMCV were also observed in intestinal contents of crested porcupines by IEM. RT-PCR end point protocol [11] detected EMCV both in organs and in cell culture lysates (Table 2).

**Table 2:** Results of virological examination on samples collected in EMCV outbreak

Species^(a)^	Samples^(b)^	Cell culture	IEM^(c)^	RT-PCR^(c)^
*Macaca sylvanus* (4)	Myocardium (4) Brain (2) Lung (2) Liver (2) Spleen (1)	+ + + + +	+ NA NA NA NA	+ + + + +
*Hystrix cristata* (9)	Myocardium (9) Brain (8) Liver (9) Intestinal content (9)	+ + + NA	+ NA NA + (3/9)	+ + + + (4/9)

^(a)^In parentheses the number of examined animals. ^(b)^In parentheses the number of samples analysed. ^(c)^In parentheses the number of positive samples/total analysed samples. *NA* not analysed

**Fig. 3:**
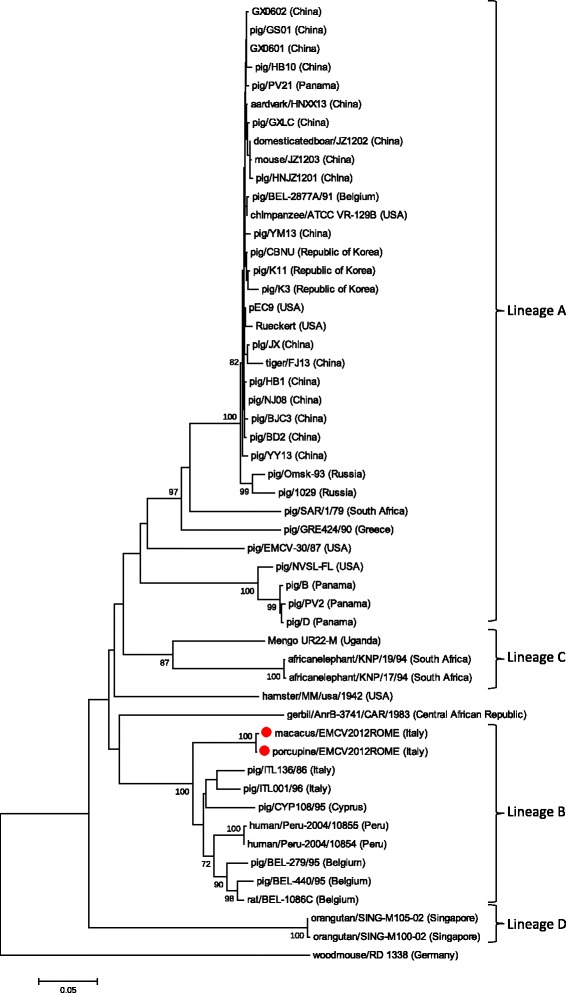
Phylogenetic tree of the VP1 coding sequences of EMCV Italian strains isolated in 2012. The macaque and porcupine EMCV strains highlighted with red dots, clusterize in a unique lineage (Lineage B) with other Italian and European strains found in GenBank database. The very high bootstrap value (100) registered between them confirms that only one EMCV strain is circulating in Italy, capable of infecting different animal species. The subdivision in lineages has been made in accordance with previous works [4, 5]

Nucleotide BLAST results suggested highest match of 91–96% sequence with the 3’ conserved end of 3D sequence DQ835185-2 in GenBank database, identifying the EMCV species. With regard to virus typing, the VP1 coding sequences show that the two isolated EMCV strains from macaque and porcupine clusterize in the unique lineage B with other Italian and European strains found in database with a high bootstrap value (100) (Fig. 3).

A rat disinfection program is routinely applied once a week in the Centre. To reduce a probable source of infection for the susceptible species, a supplementary rat control program was adopted also in those areas used for animal food storage and preparation. Biosecurity measures were also applied in the Centre as far as possible.

## Conclusions

This case describes an EMCV outbreak in captive Barbary macaques and crested porcupines in Italy, 5 years after the one reported in lemurs in another Italian open-field zoo park [3]. It confirms the susceptibility of non-human primates to the EMCV infection. On the contrary, EMCV has rarely been isolated before from crested porcupine [12, 13].

EMCV infection may often be clinically non-evident, but on the contrary, may be also characterized by an extreme virulence, lethality and severity [3]. Thus, when sudden death of non-human primates occurs without obvious symptoms and a severe myocarditis is observed at necropsy EMCV should always be included in differential diagnosis. The infection is typically reported during cold months [14] as indicated by the time period of this case.

No information is available on the possibility of EMCV having been introduced by another species in the Rescue Centre. During the outbreak three rats (two *Rattus norvegicus* and one *Rattus rattus*) and two country mice (*Mus musculus*) were found dead in the area close to the macaques enclosure but nor lesions nor EMCV were detected. Dormice [10] and squirrels live in the area, but none of these were found dead. In a past serosurvey conducted in Tuscany in 1995, specific EMCV-antibodies were detected in a wide range of species among domestic and wild animals [15] which showed that the virus was actively circulating.

Unfortunately, to avoid causing unnecessary and potentially damaging stress, no blood samples were collected from the surviving animals to verify their seropositivity or their status of persistently infected, but EMCV is known to be a potential issue for other zoological park animals as it may involve several species [2, 3, 7, 8].

The results of this report indicate that EMCV is responsible of myocarditis and sudden asymptomatic death both in Barbary macaque and crested porcupine. Phylogenetic analysis confirms that only one EMCV strain is circulating in Italy, capable of infecting different animal species.

No human cases were observed, but given the zoonotic potential of EMCV, these findings are of importance in the context of animal-human interface.

## Abbreviations

BHK21: Baby Hamster Kidney
BLAST: Basic local alignment search tool
EMCV: Encephalomyocarditis virus
IEM: Immunoelectron microscopy
IZSLER: Istituto Zooprofilattico Sperimentale della Lombardia e dell’ Emilia Romagna
IZSLT: Istituto Zooprofilattico Sperimentale del Lazio e della Toscana
PTA: Phosphotungstic acid
RT-PCR: Reverse Transcriptase-Polymerase Chain Reaction
WWF: World wide fund

## References

[1] Phillips A, Dauber M, Groth M, Schirrmeier H, Platzer M, Krumbholz A (2012). Isolation and molecular characterization of a second serotype of the encephalomyocarditis virus. Vet Microbiol.

[2] Yeo DS-Y, Lian JE, Fernandez CJ, Lin Y-N, Liaw JC-W, Soh M-L (2013). A highly divergent Encephalomyocarditis virus isolated from nonhuman primates in Singapore. Virol J.

[3] Canelli E, Luppi A, Lavazza A, Lelli D, Sozzi E, Moreno Martin AM (2010). Encephalomyocarditis virus infection in an Italian zoo. Virol J.

[4] Van Sandwyk JHT, Bennett NC, Swanepoel R, Bastos ADS (2013). Retrospective genetic characterisation of *Encephalomyocarditis viruses* from African elephant and swine recovers two distinct lineages in South Africa. Vet Microbiol.

[5] Koenen F, Vanderhallen H, Dickinson ND, Knowles NJ (1999). Phylogenetic analysis of European encephalomyocarditis viruses: comparison of two genomic regions. Arch Virol.

[6] Lin W, Liu Y, Cui S, Liu H (2012). Isolation, molecular characterization, and phylogenetic analysis of porcine encephalomyocarditis virus strain HB10 in China. Infect Genet Evol.

[7] Gaskin JM, Jorge MA, Simpson CF, Lewis AL, Olson JH, Schobert EE, et al. The tragedy of encephalomyocarditis virus infection in zoological parks of Florida. Proceedings American Association of Zoo Veterinarians. 1980; 1–7.

[8] Wells SK, Gutter AE, Soike KF, Baskin GB (1989). Encephalomyocarditis virus: epizootic in a zoological collection. J Zoo Wildl Med.

[9] Oberste MS, Gotuzzo E, Blair P, Nix WA, Ksiazek TG, Comer JA (2009). Human febrile illness caused by encephalomyocarditis virus infection. Peru Emerg Infect Dis.

[10] Amaddeo D, Cardeti G, Autorino GL. Isolation of encephalomyocarditis virus from dormice (*Myoxus glis*) in Italy. J Wildl Dis. 1995;31(2):238-42. 10.7589/0090-3558-31.2.238 8583644

[11] Bakkali Kassimi L, Gonzague M, Boutrouille A, Cruciere C (2002). Detection of encephalomyocarditis virus in clinical samples by immunomagnetic separation and one-step RT-PCR. J Virol Methods.

[12] Heredia N, Merino N, Santos O (1982). Reporte de un brote de Encefalomiocarditis en simios y puercoespines (*Hystrix cristata*). Rev Cub Cienc Vet.

[13] Ramos JR, Luya MJ (1983). Encefalomyocarditis (EMC) viral, reproducciòn experimental en Cerdos Lactantes. Rev Cub Cienc Vet.

[14] Murname TG, Beran GW (1981). Encephalomyocarditis. CRC Handbook Series in Zoonoses, Section B, vol.2. Viral Zoonoses.

[15] Cardeti G, Pattono D, Forletta R, Paladini A, Maggiori F, Luberti M, et al. Virus dell'Encefalomiocardite: diffusione dell’infezione in specie animali selvatiche e domestiche ed in gruppi selezionati di popolazione umana. In: Atti Secondo Convegno sui programmi di ricerca finalizzata e corrente degli Istituti Zooprofilattici Sperimentali: 25–26 June 1997, Rome, Italy. Rome: Istituto Superiore di Sanità; 1997. p. 60.

